# Transcriptome Analysis and CFEM Gene Overexpression in *Metschnikowia bicuspidata* Under Hemocyte and Iron Ion Stress

**DOI:** 10.3390/pathogens14070691

**Published:** 2025-07-14

**Authors:** Bingnan Zuo, Xiaodong Li, Ji Zhang, Bingyu Li, Na Sun, Fang Liang

**Affiliations:** 1 Key Laboratory of Livestock Infectious Diseases in Northeast China, Ministry of Education, Key Laboratory of Zoonosis, Shenyang Agricultural University, Shenyang 110866, China; 2021200171@stu.syau.edu.cn (B.Z.); zj973318902@gmail.com (J.Z.); 2 College of Aquaculture and Life Sciences, Dalian Ocean University, Dalian 116023, China; libingyu@dlou.edu.cn; 3 Panjin Guanghe Crab Industry Co., Ltd., Panjin 124000, China; sunna0911@163.com (N.S.); 15842703137@163.com (F.L.)

**Keywords:** CFEM, hemocyte stress, *Metschnikowia bicuspidata*, transcriptome

## Abstract

The “milky disease” in Chinese mitten crabs (*Eriocheir sinensis*), caused by *Metschnikowia bicuspidata*, poses significant threats to aquaculture, though its pathogenic mechanisms remain poorly understood. This study employs transcriptomic sequencing to analyze gene expression changes in *Metschnikowia bicuspidata* under hemocyte challenge, iron overload (1 mmol/mL), and combined stress, with functional validation through Common in Fungal Extracellular Membrane (CFEMgene) overexpression strains. Key findings reveal that (1) hemocyte challenge activated base excision repair (−log_10_[P] = 7.58) and ribosome biogenesis pathways, indicating fungal adaptation through DNA repair and enhanced protein synthesis to counter host immune attacks (e.g., ROS-mediated damage). (2) Iron overload induced glutathione metabolism and pentose phosphate pathway enrichment, demonstrating mitigation of ferroptosis through NADPH/GSH antioxidant systems and autophagy/proteasome coordination. (3) Under combined stress, ribosome biogenesis (−log_10_[P] = 1.3) and non-homologous end-joining pathways coordinated DNA repair with stress protein synthesis, complemented by vacuolar V-ATPase-mediated iron compartmentalization. (4) CFEM genes showed significant upregulation under hemocyte stress, with overexpression strains exhibiting enhanced biofilm formation (35% increased MTT cytotoxicity) and infectivity (40% higher infection rate), confirming CFEM domains mediate pathogenesis through iron homeostasis and virulence factor production. This work elucidates how *M. bicuspidata* employs metabolic reprogramming, oxidative stress responses, and CFEM-mediated iron regulation to establish infection, providing critical insights for developing targeted control strategies against milky disease.

## 1. Introduction

The Chinese mitten crab (Eriocheir sinensis), a decapod crustacean of the family Grapsidae, represents a commercially vital aquaculture species in China [1]. In 2023, Chinese mitten crab production reached 888,600 tons [2]. In the winter of 2018, an outbreak of “milky disease” in Panjin devastated overwintering juvenile and broodstock crabs; the morbidity rate was over 80%, and the disease mortality rate was nearly 100%, characterized by whitened body coloration, reduced mobility, and the accumulation of milky fluid beneath the carapace [3]. This has become the most important disease of river crabs in Liaoning, Zhejiang, and Hebei, affecting the stability and healthy development of the river crab industry [4,5,6,7]. Microscopic analysis confirmed hemolymph emulsification with distinctive opaque whitening. Pathogen isolation and sequencing identified *Metschnikowia bicuspidate* (*M*. *bicuspidata*) as the causative agent [8,9]. *M*. *bicuspidata* is an opportunistic fungal pathogen that thrives under specific host conditions [10]. With rising incidence rates threatening northern crab aquaculture sustainability, and given the absence of effective therapeutics or mechanistic studies on infection pathways, this investigation was undertaken to address critical knowledge gaps [3].

As a pathogenic fungus, its virulence mechanisms primarily rely on extracellular secreted proteins. Glycosylphosphatidylinositol (GPI)-anchored modification represents a prevalent post-translational modification for membrane-associated proteins in eukaryotes [11]. GPI anchors mediate protein localization to the outer leaflet of plasma membranes [12]. In yeast, GPI-anchored proteins are essential for the viability and maintenance of cellular morphology. These GPI-modified cell wall proteins critically regulate wall biosynthesis and structural integrity through coordinated interactions with wall components and other GPI-anchored proteins. The CFEM (Common in Fungal Extracellular Membrane) domain, characterized by eight conserved cysteine residues [13], functions as a cellular sensor through redox-active thiol groups [14,15]. CFEM domains serve as crucial mediators of fungal pathogenicity [16], influencing cell wall stability and infection progression [17,18]. Phylogenetic analysis of 363 CFEM domains across 64 pathogenic fungal genera by Zhang revealed conserved 60 amino acid modules with quantitative variation between species. Notably, pathogenic strains in *Ascomycota*, *Basidiomycota*, and *Saccharomycetaceae* exhibited significantly more CFEM domains than non-pathogenic counterparts, suggesting a positive correlation between CFEM copy number and fungal virulence potential [19].

To elucidate host/pathogen interactions during milky disease progression, we employed next-generation sequencing (NGS) to systematically profile hemolymph transcriptomic responses in infected crabs and parallel genomic adaptations of *M. bicuspidata* under host-mimicked stress. This dual-pronged approach enables deciphering crustacean immune defenses against fungal invasion through differential gene expression analysis and delineating pathogen virulence mechanisms via fungal genomic plasticity assessment. The integrated findings establish a systematic framework for developing evidence-based therapeutic interventions and prophylactic measures, ultimately informing targeted disease management strategies to ensure the sustainable development of crustacean aquaculture.

## 2. Method and Materials

### 2.1. Experimental Strains and Animals

The strain *M. bicuspidata* (LNPJ 1214) used in this experiment and juvenile Chinese mitten crabs were obtained from Panjin Photosynthesis R&D Center. The 600 juvenile Chinese mitten crabs weighed 25 ± 5 g and were maintained in aquaculture tanks under the following controlled conditions: dissolved oxygen (DO) 6.0 ± 0.5 mg/L, pH = 7, salinity 1‰, and temperature 28 °C. After a 7-day acclimation period, experimental specimens demonstrating intact carapaces, complete appendages, and vigorous motility were selected for subsequent procedures.

### 2.2. Hemocyte Stress

#### 2.2.1. *M. bicuspidata* Culture

Preserved fungal strains were streaked on YPD (1% yeast extract, 2% peptone, 2% glucose) solid medium and incubated at room temperature for 24 h. Single colonies were selected for verification through colony PCR before being transferred to a YPD broth medium for overnight culture. The overnight culture was centrifuged (3000× *g*, 10 min) to pellet cells. After supernatant removal, the pathogen pellet was resuspended in sterile distilled water and adjusted to 1 × 10^10^ CFU/mL using McFarland standards.

#### 2.2.2. Hemocyte Acquisition

Healthy juvenile Chinese mitten crabs were selected for hemolymph collection. A 1 mL syringe preloaded with 500 μL anticoagulant solution (containing 0.774 g trisodium citrate, 1.97 g NaCl, 2.07 g glucose, and 0.374 g EDTA in 100 mL deionized water, pH 7.0) was used for hemolymph extraction. Following hemolymph aspiration (500 μL), the sample was transferred to a 50 mL centrifuge tube and centrifuged at 1000 rpm for 5 min at 4 °C. The supernatant was carefully discarded. The pellet was washed twice with phosphate-buffered saline (PBS) through sequential resuspension/centrifugation cycles (1000 rpm, 5 min). After final supernatant removal, cells were resuspended in TC-100 medium supplemented with 10 mL saturated NaCl and 1% penicillin/streptomycin solution, with final cell density adjusted to 2 × 10^8^ CFU/mL using a hemocytometer.

#### 2.2.3. Hemocyte Co-Culture Assay

*M.bicuspidata* cells were co-cultured with crab hemocytes at a 1:50 (fungus/hemocyte) ratio in triplicate biological replicates. Experimental groups included *M. bicuspidata* control (sterile medium, M/CK), hemocyte stress control (MB/T1), Fe^3+^ ion control (1 mM FeCl_3_, MF/T2), and co-stress group (MBF/T3). Cultures were maintained at 28 °C for 8 h in a humidified incubator. Post-incubation, samples were centrifuged (3000× *g*, 10 min) to pellet cells, flash-frozen in liquid nitrogen for 12 h, and stored at −80 °C until RNA extraction.

#### 2.2.4. Total RNA Extraction and Quality Assessment

Total RNA was extracted by the bead milling and hot phenol method, concentration and purity were measured by nanodrop using Thermo Scientific NanoDrop 2000 (Thermo Scientific, Waltham, MA, USA), and the integrity was measured by agarose electrophoresis for RNA or 2100 using an Agilent 2100 Bioanalyzer. RNA 6000 Nano kit 5067-1511 (Agilent Technologies Inc., Santa Clara, CA, USA).

#### 2.2.5. Library Preparation and Sequencing

High-quality RNA samples were sent to Personal Biotechnology Co., Ltd. (Shanghai, China) for library construction using the NEBNext^®^ Ultra™ RNA Library Prep Kit (New England Biolabs, Ipswich, MA, USA). Sequencing was performed on an Illumina HiSeq 2000 platform with 150-bp paired-end reads. Raw sequencing data (FASTQ format) were generated through base calling of fluorescent signals using Illumina’s proprietary software (v.1.8.2), with quality filtering applied to remove adaptor sequences and low-confidence reads (Phred score < Q30).

#### 2.2.6. Library Construction and Quality Control

Total RNA (≥1 μg) was processed using the NEBNext Ultra II Directional RNA Library Prep Kit (NEB, E7760, New England Biolabs, Ipswich, MA, USA) for strand-specific library preparation. Polyadenylated mRNA was enriched via Oligo (dT) magnetic bead selection (NEB, E7490, New England Biolabs, Ipswich, MA, USA) and fragmented using divalent cations under elevated temperature (94 °C, 8 min). First-strand cDNA synthesis was performed with random hexamer primers, followed by second-strand generation using DNA Polymerase I. Double-stranded cDNA was purified (AMPure XP Beads, Beckman Coulter, A63881, Brea, CA, USA), end-repaired, and adenylated at 3′ termini for Illumina adapter ligation (NEBNext Adaptor, NEB, E7335,New England Biolabs, Ipswich, MA, USA). Size selection (400–500 bp) was achieved through dual AMPure XP bead purification (0.6× and 1.2× ratios). Library amplification involved 12 PCR cycles with NEBNext Universal Primers, followed by final bead-based purification. Library quality was assessed using an Agilent 2100 Bioanalyzer with High Sensitivity DNA Kit (5067-4626, Santa Clara, CA, USA), verifying insert size distribution (425 ± 25 bp) and adapter dimer elimination. Absolute quantification was performed using the Quant-iT PicoGreen dsDNA Assay (Invitrogen, P7589, Waltham, MA, USA) on a Quantifluor-ST fluorometer (Promega, E6090, Madison, WI, USA), while functional library concentration was determined via qPCR (StepOnePlus System, Thermo Fisher, Waltham, MA, USA) with the KAPA Library Quantification Kit (Roche, KK4824, Basel, Switzerland). Indexed libraries were normalized to 4 nM based on qPCR values, pooled equimolarly, and sequenced in PE150 mode on an Illumina NovaSeq 6000 platform (Illumina, SY-402-1001, San Diego, CA, USA) following standard denaturation and dilution protocols.

#### 2.2.7. Transcriptome Annotation

The unigenes obtained from the Trinity system splicing were subjected to ORF prediction and BLASTx similarity search. Then, they were compared with NR, Swiss-Prot (http://www.ebi.ac.uk/uniprot/, accessed on 3 July 2023), Pfam (http://pfam.xfam.org/, accessed on 9 July 2023), GO (Gene Ontology) (http://www.geneontology.org/, accessed on 16 July 2023), and KEGG (Kyoto Encyclopedia of Genes and Genomes) database for comparative annotation (http://www.genome.jp/kegg/, accessed on 25 July 2023), E-Value < 10^−5^.

#### 2.2.8. Reference Genome Alignment and Quantification

Genome indexing was performed using HISAT2 (v2.1.0) with default parameters. High-quality paired-end reads were aligned to the reference genome using HISAT2 in end-to-end mode, with alignment metrics recorded using SAMtools (v1.9). Gene-level read counts were quantified via HTSeq-count (v0.9.1) in union mode, excluding ambiguous reads overlapping multiple genes. Expression values were normalized using Fragments Per Kilobase of transcript per Million mapped reads (FPKM) to enable cross-sample comparisons. Differential expression analysis between experimental groups was conducted using DESeq2 (v1.38.3) with Benjamini–Hochberg multiple testing correction. Significantly differentially expressed genes (DEGs) were identified using thresholds of |log_2_(fold change)| > 1 and adjusted *p*-value < 0.05, following shrinkage estimation with the apeglm algorithm.

#### 2.2.9. CFEM Gene Overexpression

**Figure 1:**
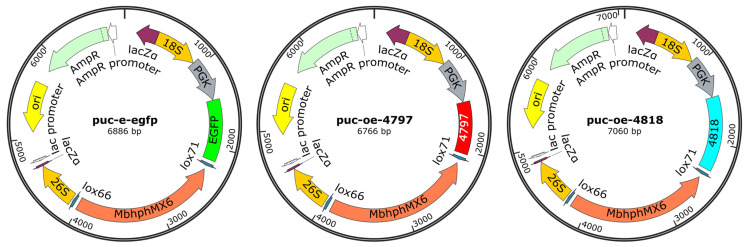
The CFEM overexpression plasmid.

The pUC-OE plasmid backbone was linearized using restriction enzymes (FastDigest EcoRI/XhoI, Thermo Scientific, Waltham, MA, USA) (Figure 1). CFEM domains were PCR-amplified with primers (designed by Oligo 7/version 7.56) listed in Table 1 using Q5 High-Fidelity DNA Polymerase (NEB, M0491) under cycling conditions, 2× Taq PCR MasterMix Ⅱ 12 μL., up and down primers 1 μL (10 μM (Table 1)), 1 μL DNA, 10 μL ddH_2_O. Amplified fragments were purified (Monarch PCR Cleanup Kit, T1020) and cloned into linearized pUC-OE via Gibson Assembly^®^ Master Mix (NEB, E2611). The recombinant plasmids were transformed into E. coli DH5α competent cells (Takara, 9057, Shizuoka, Fuji City, Japan) and plated on LB agar with ampicillin (100 μg/mL). Positive clones were verified by colony PCR and Sanger sequencing. Validated plasmids were extracted (GeneJET Plasmid Miniprep Kit, K0502), and overexpression cassettes were amplified using PrimeSTAR Max DNA Polymerase (Takara, R045A). Purified fragments (ethanol precipitation with 0.3 M sodium acetate) [20] were electroporated into *M. bicuspidata* competent cells (Bio-Rad Gene Pulser Xcell, 1.5 kV, 200 Ω, 25 μF), followed by recovery in YPD medium at 28 °C for 48 h.

#### 2.2.10. Validation of Overexpression Strains and Transcriptomic Profiling

Total RNA was isolated from yeast transformants using bead-beating homogenization coupled with hot phenol-chloroform extraction [21]. First-strand cDNA synthesis was performed using the HiScript III 1st Strand cDNA Synthesis Kit (Vazyme Biotech, R312, Nanjing, China) with gene-specific primers (designed by Oligo 7/version 7.56) (Table 2). Quantitative PCR analysis was conducted in triplicate technical replicates using the ChamQ Universal SYBR qPCR Master Mix (Vazyme Biotech, Q711, Nanjing, China) under optimized cycling conditions as follows: 2 × ChamQ Universal SYBR qPCR Master Mix 10 μL, up and down primers 0.5 μL (10 μM), cDNA 1 μL, and 8 μL DEPC H_2_O. The endogenous reference gene β-actin demonstrated stable expression across experimental conditions (CV < 0.5). Relative gene expression levels were calculated using the comparative 2^−ΔΔCt^ method (*p* < 0.05).

#### 2.2.11. Physiological Characterization of Overexpression Strains

Divided into the *M. bicuspidata* group M were the 4818 gene overexpression group 4818g, the 4797 gene overexpression group 4797g, the Fe ion group (MF, 4818gF, 4797gF), and the hemocyte group (MB, 4818gB, 4797gB), and the co-stress group (MBF, 4818gBF, 4797gBF), the hemocyte B, the hemocyte with Fe ion BF group with three biological replicates per group.

Growth Kinetics: Overnight cultures were inoculated (1% v/v) into fresh YPD broth and incubated at 28 °C with 160 rpm agitation. Optical density at 600 nm was measured every 4 h using a microplate reader (BioTek Synergy H1) over a 48-h period, with triplicate biological replicates per strain.

Senna Assay for Biofilm Biomass: Saline-washed suspensions of each strain were prepared by incubation on YPD plates for 24 h at 28 °C. The turbidity of each suspension was adjusted to 3 × 10^7^ cfu/mL with YPD supplemented with glucose (final concentration, 8%). Then, 1 mL of suspension was inoculated into polystyrene tubes containing 9 mL of SDB, and each well was inoculated with 200 uL of yeast cell suspension and incubated for 24 h at 28 °C. The suspension was then washed with phosphate buffer. The microtiter plate was then washed four times with phosphate buffer, stained with 1% Senna red, aspirated, and read spectrophotometrically at 490 nm using a microtitre plate reader. The percentage transmittance (% Tbloc) of each test sample was subtracted from the % T value of the reagent blank to obtain a measurement of the amount of temporal blockage through the wells (% Tbloc) [22].

Toxicity was detected by the MTT method [23] where 100 uL of the hemocyte suspensions of the Chinese mitten crab and *M. bicuspidata* were added at a ratio of 1:50 in a 96-well culture plate for the parallel determination. The blood cell and *M.bicuspidata* controls were prepared at the same time. Three replicates were set up for each sample and cultured in an incubator at 28 °C for 24 h. At the end of culture, MTT was added for staining using a tetramethyl azole blue (MTT) kit (UW Genetics, China) according to the instructions, and the absorbance of the samples at 570 nm (Optical density (OD)) was determined to calculate the cell proliferation rate and inhibition rate (toxicity). According to the toxicity formula

Toxicity = 1 − experimental group/blank group

The higher the toxicity of the strain, the lower the absorbance.

Determination of Flocculation Rate of Overexpression Strains: Take a single colony of 5 strains of bacteria after activation on the plate and add it into 5 mL of YPD liquid medium (in which 30 μg/mL G418 was added to the overexpression strains) and incubate it at 28 °C overnight, then add it into 100 mL of YPD liquid according to the inoculum quantity of 1% and incubate it at 28 °C for 48 h. Centrifuge the organisms at 5000 rpm for 5 min to collect the bacteria, and then wash them with PBS buffer two times, resuspend them, and use a hemocytometer plate to count the number of cells. After resuspension, the bacterial fluid with a concentration of 2 × 10^8^ CFU/mL was counted by using a hemocyte counting plate, vortexed and oscillated for 1 min, and then added into a transparent test tube for resting, and the supernatant was taken to detect the OD600 absorbance value every 20 min until 180 min, and 3 parallels were determined in each group.

#### 2.2.12. Overexpression Strain Back-Sense Experiment

The juvenile Chinese mitten crabs, weighing 10 ± 2 g, were selected and divided into blank, 4797 overexpression, 4818 overexpression, and added Fe ions groups of 30 crabs in each group, and three biological replicates were carried out in each group. The experiment began one week after the river crabs were temporarily reared in a 100 L tank. The crabs were fed a commercial diet at 2% of their body weight daily. The water temperature during the experiment was 15 ± 2 °C. The water was fully changed after each day’s feeding. After the water change, the resuspension solution was added according to the grouping, resulting in a final bacterial concentration of 5 × 10^5^ CFU/mL. Then, the Fe ion group was added, resulting in a final Fe ion concentration of 1 mmol/L.

#### 2.2.13. Statistical Analysis

Chi-square, Fisher’s exact tests, and the Kruskal–Wallis nonparametric test were performed using SPSS (v21.0) to determine significant relationships between groups statistically (*p* < 0.05), while data visualization was conducted with OriginPro (v9.9).

## 3. Result

### 3.1. Sample Quality Control (QC)

**Figure 2:**
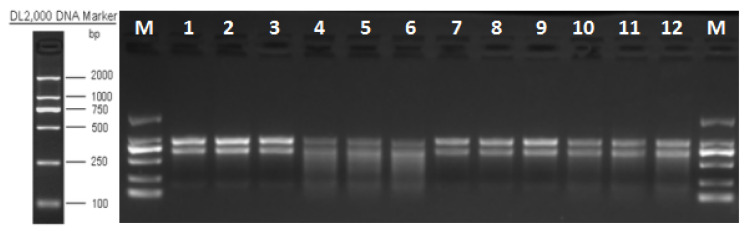
RNA electropherogram. Note: 1–3 *M. bicuspidata* control (sterile medium, M/CK), 4–6 hemocyte stress control (MB/T1), 7–9 Fe^3+^ ion control (1 mM FeCl_3_, MF/T2), and 10–12 co-stress group (MBF/T3).

The results showed that the integrity of RNA extraction was good in all groups, and after sequencing quality control, the percentage of Q30 bases in each sample was greater than 93.74% for subsequent analysis (Figure 2, Table 3).

### 3.2. Sample Correlation Analysis

**Figure 3:**
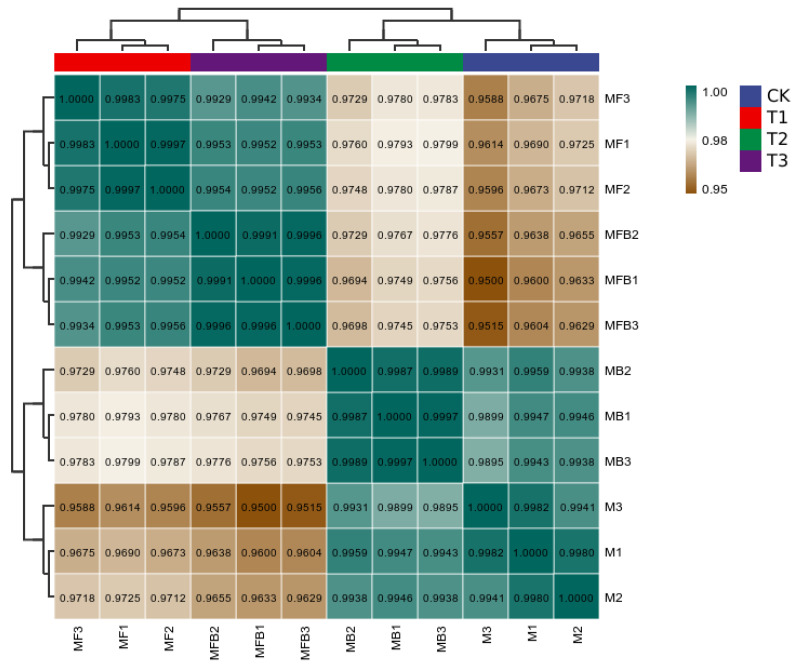
Correlation analysis of data for each group of the experiment. Note: *M. bicuspidata* control (sterile medium, M/CK), hemocyte stress control (MB/T1), Fe^3+^ ion control (1 mM FeCl_3_, MF/T2), and co-stress group (MBF/T3).

Pearson’s correlation coefficient (R) is used as an index to assess the biological correlation between replicates. The closer the R2 is to 1, the stronger the correlation between two replicates, which should not be less than 0.8. In the present data, the R-value is greater than 0.95, and the data correlation is high (Figure 3).

### 3.3. Differential Gene Count

**Figure 4:**
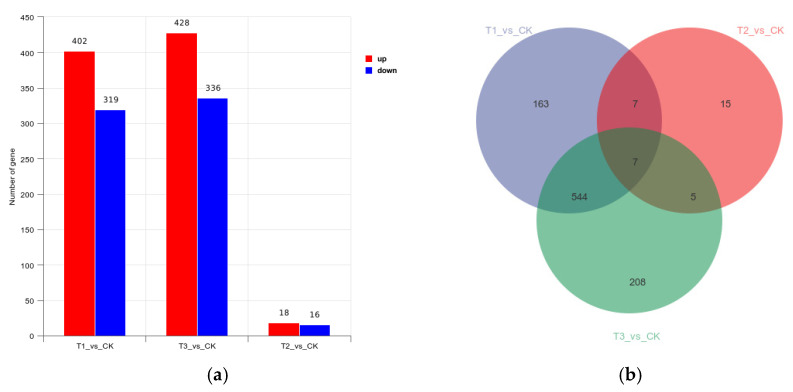
(**a**) Differential Gene Expression Volcano Plot; (**b**) Differential Gene Expression Venn Plot. Note: CK is the *M. bicuspidata* control, T1 is the hemocyte stress group, T2 is the Fe ion stress group, and T3 is the co-stress group.

A total of 747 differential genes were compared in the hemocyte stress group, of which 428 were up-regulated and 319 were down-regulated; 34 differential genes were compared in the Fe ion stress group, of which 18 were up-regulated and 16 were down-regulated; and 764 differential genes were compared in the co-stress group, of which 428 were up-regulated and 336 were down-regulated. The three groups had only seven common differential genes (Figure 4).

**Figure 5:**
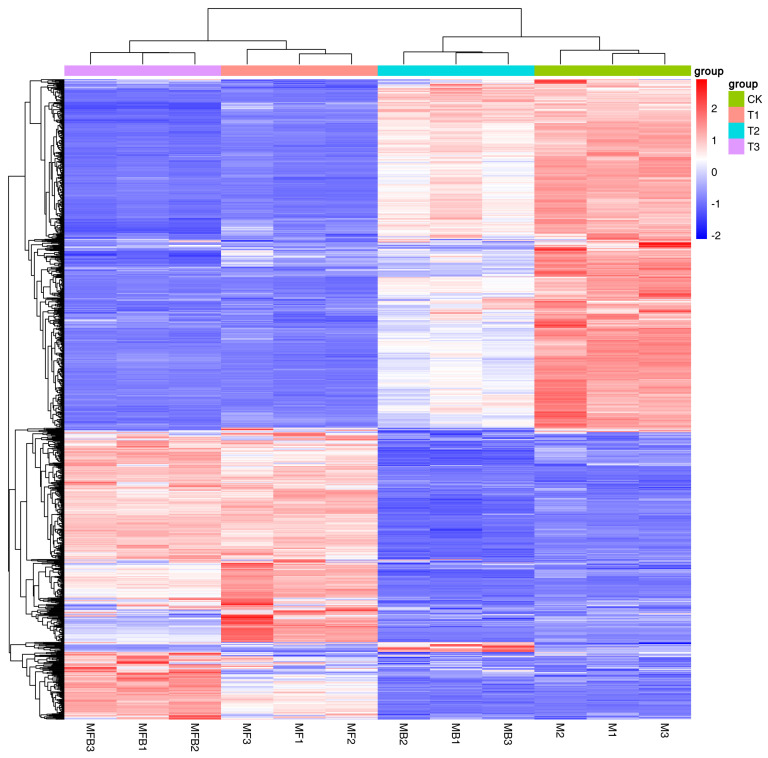
Heat map of gene expression. Note: *M. bicuspidata* control (sterile medium, M/CK), hemocyte stress control (MB/T1), Fe^3+^ ion control (1 mM FeCl_3_, MF/T2), and co-stress group (MBF/T3).

As illustrated in Figure 5, the replicate expression trend of the same treatment group exhibited consistency, indicating the reproducibility of the experiment and the reliability of the data. The hemocyte stress group exhibited significant up-regulation of certain genes, as evidenced by the heat map. In the Fe ion stress group, several genes demonstrated a moderate decrease in expression, which may be associated with the inhibition of metal ion homeostatic regulatory genes, such as those involved in iron carrier synthesis. The elevated expression levels of select genes in the co-stress group suggest that the combined stress may elicit synergistic (e.g., the superposition of dual stress pathways) or antagonistic (e.g., cross-regulation leading to the inverse expression of certain genes) effects. The expression trends of MFB1 and MFB2/MFB3 in specific genes exhibited slight variations, which necessitate further analysis and validation (Figure 5).

### 3.4. Comparison of Hemocyte Stress Gene Expression

#### 3.4.1. Differentially Expressed Genes

**Figure 6:**
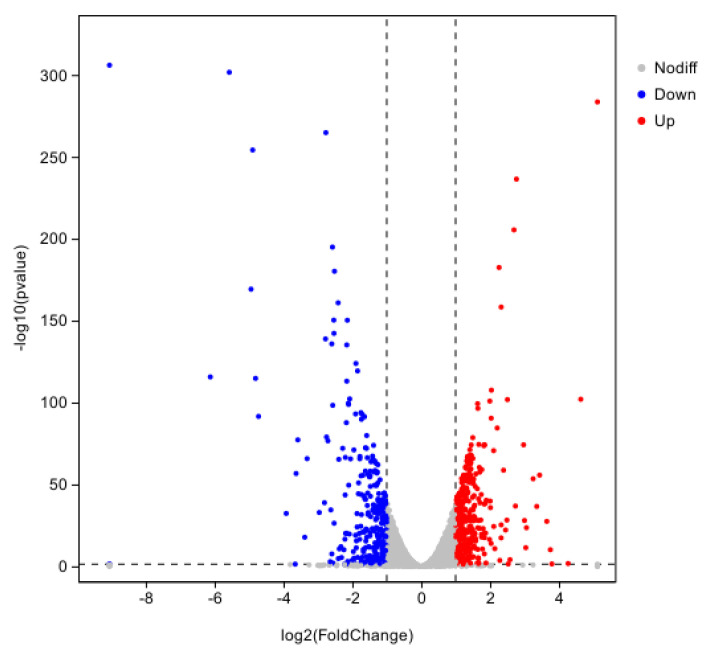
Volcano plot of differential gene expression for 8 h of hemocyte stress.

The hemocyte stress group was screened at |log2FC| ≥ 1.5 and *p* < 0.01, and a total of 747 DEGs were obtained, of which 428 were up-regulated genes and 319 were down-regulated genes (Figure 6).

#### 3.4.2. GO Analysis of Differentially Expressed Gene DEGs

**Figure 7:**
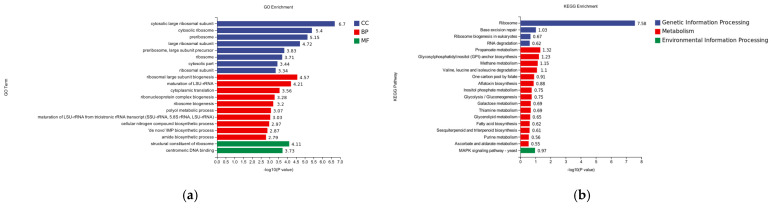
(**a**) GO classification of differentially expressed genes in hemocytes stressed for 8 h; CC is Cellular Component, BP is Biological Process, and MF is Molecular Function. (**b**) Analysis of KEGG enrichment of differential genes for 8 h of hemocyte stress.

In the differential gene GO enrichment analysis of the hemocyte stress group, a total of 20 significantly enriched gene ontology entries were identified, covering the three major categories of Cellular Component (CC), Biological Process (BP), and Molecular Function (MF). The following entries were determined to be of significant interest within the cellular component (CC): Cytosolic large ribosomal subunit (−log10 = 6.7). The analysis revealed a significant enrichment of the cytosolic ribosome (log10P = 5.4) BP for ribosome large subunit biogenesis (ribosome biosynthesis). The analysis revealed a statistically significant enrichment of molecular functions associated with bioprocesses, with two unique functions identified. The first function, the structure constituent of the ribosome, exhibited a negative log10 probability of 4.11, while the second function, germline DNA binding, demonstrated a negative log10 probability of 3.73. In contrast, the comparison between (−log10P = 4.57) and maturation of LSU-rRNA (−log10P = 5.4) did not yield any statistically significant results (Figure 7a).

#### 3.4.3. Analysis of KEGG Enrichment of Differentially Expressed Genes

In the differential gene KEGG enrichment analysis of the hemocyte stress group, a total of 20 significantly enriched pathways were identified, covering three major categories: genetic information processing, metabolism, and environmental information processing. In the publication entitled “Genetic Information Processing”, 20 pathways were identified as being significantly enriched. These pathways encompass the domains of genetic information processing, metabolism, and environmental information processing. In the field of genetic information processing, ribosome (−logP = 7.58) and base excision repair (−logP = 1.03) exhibited significant enrichment. In the domain of metabolism, propanoate metabolism (−logP = 1.32) and glycolysis (−logP = 1.03) demonstrated notable enrichment. As indicated by the high enrichment observed, 1.32 and glycosylphosphatidylinositol (GPI)-anchor biosynthesis (logP = 7.58), there is a notable relationship between these two factors. Within the domain of environmental information processing, the MAPK signaling pathway was identified as the sole significant enrichment (Figure 7b).

### 3.5. Comparison of Iron Ion Stress Gene Expression

#### 3.5.1. Screening of Differentially Expressed Genes DEGs

**Figure 8:**
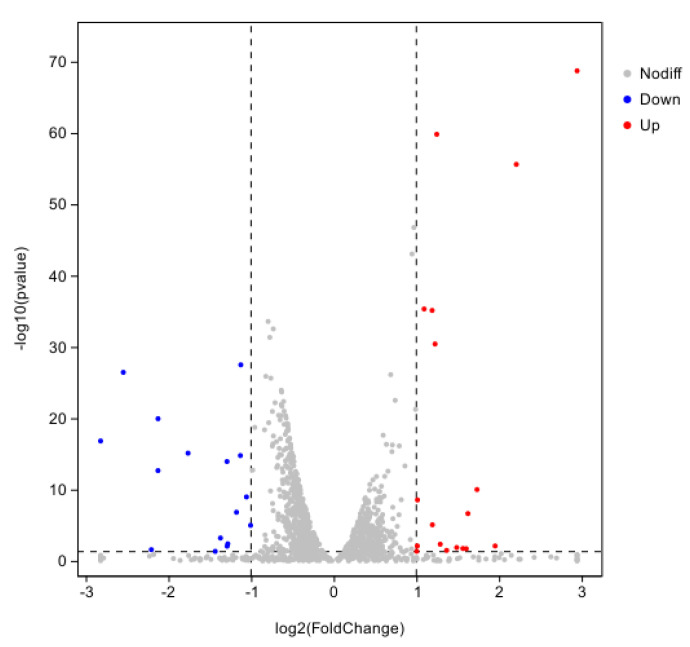
Differentially expressed genes in iron ion stress for 8 h.

Using CK as the control group and T2 as the treatment group, a total of 34 DEGs were obtained with 18 genes up-regulated and 16 genes down-regulated using DESeq2 (v.0.5.0) software with |log2FC| ≥ 1.5 and *p* < 0.01 as the screening conditions (Figure 8).

#### 3.5.2. GO Analysis of Differentially Expressed Gene DEGs

**Figure 9:**
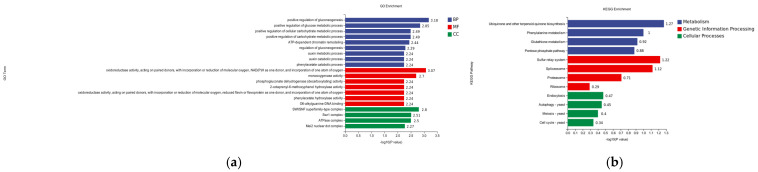
(**a**) GO classification of differentially expressed genes under iron ion stress for 8 h; CC is Cellular Component, BP is Biological Process, and MF is Molecular Function. (**b**) KEGG classification of differentially expressed genes under iron ion stress for 8 h.

The GO classification system is comprised of three primary branches: Biological Process, Molecular Function, and Cellular Component. A significant enrichment was observed for nine processes within the Biological Process category. Of particular note were positive regulation of gluconeogenesis (−log10P = 318) and the positive regulation of the glucose metabolic process (−log10P = 285), which exhibited particularly high significance. Additionally, regulation of gluconeogenesis (−log10P = 3.18) and positive regulation of glucose metabolic process (−log10P = 2). As indicated by the results of the study, Molecular Function was found to be significantly enriched in seven distinct processes, the analysis further revealed that oxidoreductase activity, acting on paired donors, incorporation or reduction of molecular oxygen, NAD(P)H, and incorporation of one atom of oxygen (−log10P = 3) were particularly notable. The Cellular Component was found to be significantly enriched for four processes, and the SWISNF superfamily-type complex (−log10P = 2.8) was found to be highly ranked (Figure 9a).

#### 3.5.3. KEGG Analysis of Differentially Expressed Gene DEGs

The results of the KEGG database analysis indicate that the differentially expressed genes were enriched in the three classes of cellular processes, genetic information processing, and metabolism (Figure 9b). As illustrated in the figure, four pathways were found to be significantly enriched for metabolism, with ubiquinone and other terpenoid/quinone biosynthesis (−log10P = 1.27) and phenylalanine metabolism (−log10P = 1) demonstrating notable enrichment. Additionally, four pathways were identified as significantly enriched for genetic information processing, with sulfur relay system (−log10P = 1.22) and spliceosome (−log10P = 1.12) demonstrating high rankings; four significantly enriched pathways for metabolism were identified, with minimal variation in significance between the groups. The following text is intended to provide a comprehensive overview of the subject matter.

### 3.6. Comparison of Gene Expression in Hemocytes Co-Stressed with Fe Ions

#### 3.6.1. Screening of Differentially Expressed Genes DEGs

**Figure 10:**
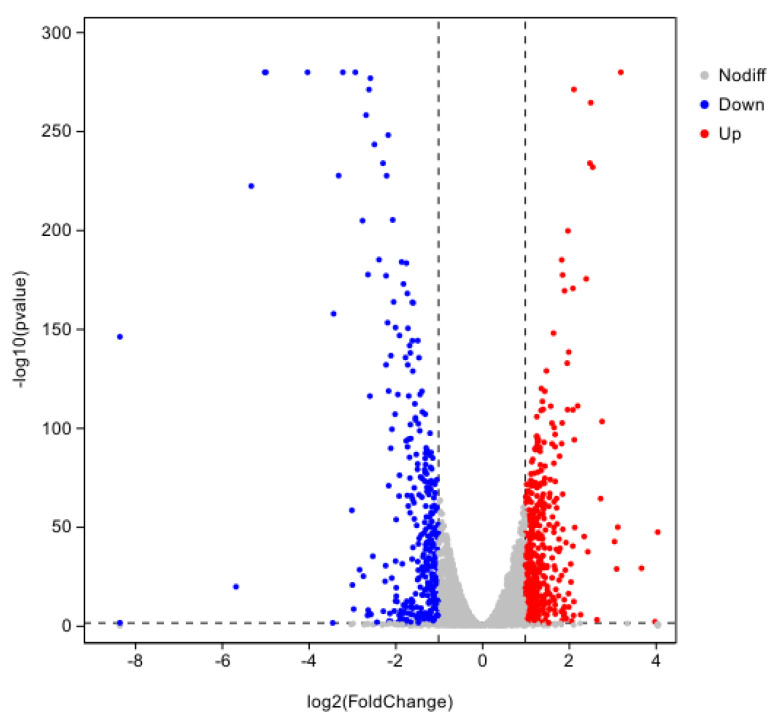
Differentially expressed genes at 8 h of co-stress.

A total of 764 differential genes, 428 up-regulated genes, and 336 down-regulated genes were compared between the hemocyte and Fe ion stress groups in the |log2FC| ≥ 1.5 and *p* < 0.01 screening conditions (Figure 10).

#### 3.6.2. GO Analysis of Differentially Expressed Gene DEGs

**Figure 11:**
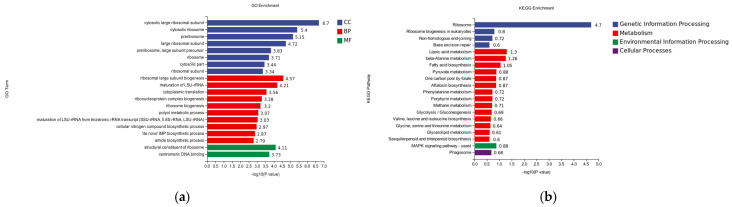
(**a**) GO classification of differentially expressed genes at 8 h of co-stress; CC is Cellular Component, BP is Biological Process, and MF is Molecular Function. (**b**) KEGG enrichment analysis of differentially expressed genes at 8 h of co-stress.

According to the results of GO enrichment analysis, there were 8 significantly enriched processes in Cellular Component, among which were cytosolic large ribosomal subunit (−log10P = 6.7), cytosolic ribosome (−log10P = 5.4), and log10P = 4.21). Only two processes were significantly enriched in molecular function: structural component of ribosome (−log10P = 4.11) and centromeric DNA binding (−log10P = 3.73) (Figure 11a).

#### 3.6.3. KEGG Analysis of Differentially Expressed Gene DEGs

According to the KEGG database analysis, genetic information processing was significantly enriched for 4 pathways, ribosome (−log10P = 4.11) and ribosome biogenesis in eukaryotes (−log10P = 0.8), and metabolism was significantly enriched for up to 14 pathways, lipoic acid metabolism (−log10P = 1.3) and beta-alanine metabolism (−log10P = 1.3). Metabolism-enriched pathways were significantly enriched for up to 14 pathways, with lipoic acid metabolism (−log10P = 1.3) and beta-alanine metabolism (−log10P = 1.26) being the most significant; cellular processes (−log10P = 1.26) were significantly enriched in one pathway; and cellular processes (−log10P = 1.26) were significantly enriched in one pathway (−log10P = 1.26); cellular processes (−log10P = 1.26) were significantly enriched in one pathway, phagosome (−log10P = 1.26) environmental information processing 1MAPK pathway yeast (−log10P = 1.26) (Figure 11b).

### 3.7. Transcriptome QPCR Validation

**Figure 12:**
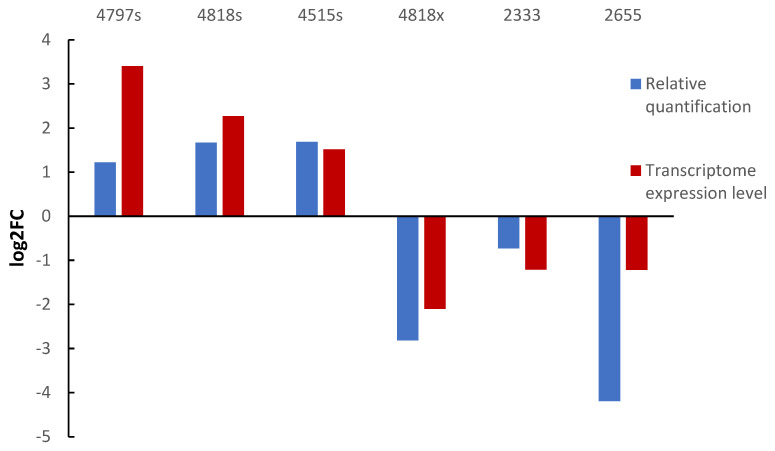
Validation of DEGs by qRT-PCR. Note: 4797s, 4818s, 4515s are the names of up-regulated genes; 4818s, 2333, 2655 are the names of down-regulated genes.

Three up-regulated and three down-regulated genes were selected for qPCR analysis to determine their relative expression. β-actin was used as the internal reference gene, and log2FC values were calculated. The log2FC values of each gene were consistent with the transcriptome expression data (Figure 12).

### 3.8. CFEM Overexpression Strain Construction

#### 3.8.1. Validation of Overexpression Strains

**Figure 13:**
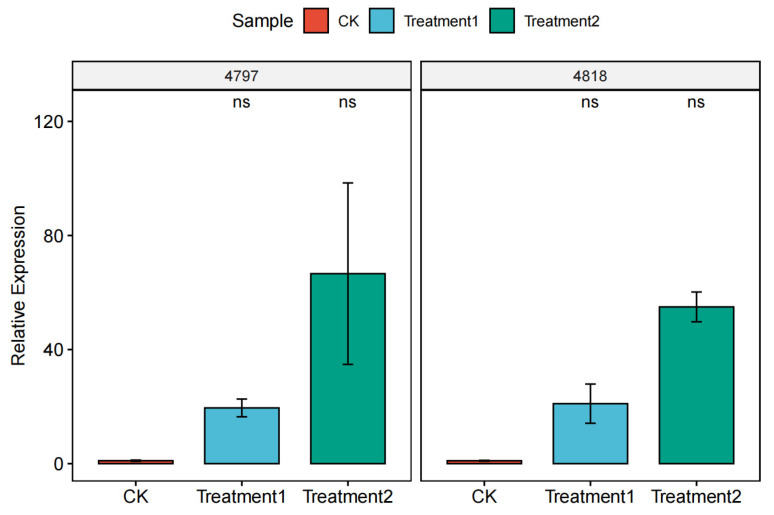
Relative expression of overexpression strains. Note; 4797: CK is the expression of the 4797 gene in *M. bicuspidata*, Treatment 1 is the expression of the 4797 gene in the 4818 gene overexpression strain, Treatment 2 is the expression of the 4797 gene in the 4818 gene overexpression strain; 4818: CK is the expression of the 4818 gene in *M. bicuspidata*, Treatment1 is the expression of the 4818 gene in the 4797 gene overexpression strain, Treatment 2 is the expression of the 4818 gene in the 4818 gene overexpression strain. “ns” means “not significant”.

The relative expression of the overexpression strains was determined by qPCR using β-actin as an internal reference gene, and the results showed that the overexpression of 4818 and 4794 genes was successful (Figure 13).

#### 3.8.2. Basic Performance Analysis of Overexpression Strains

**Figure 14:**
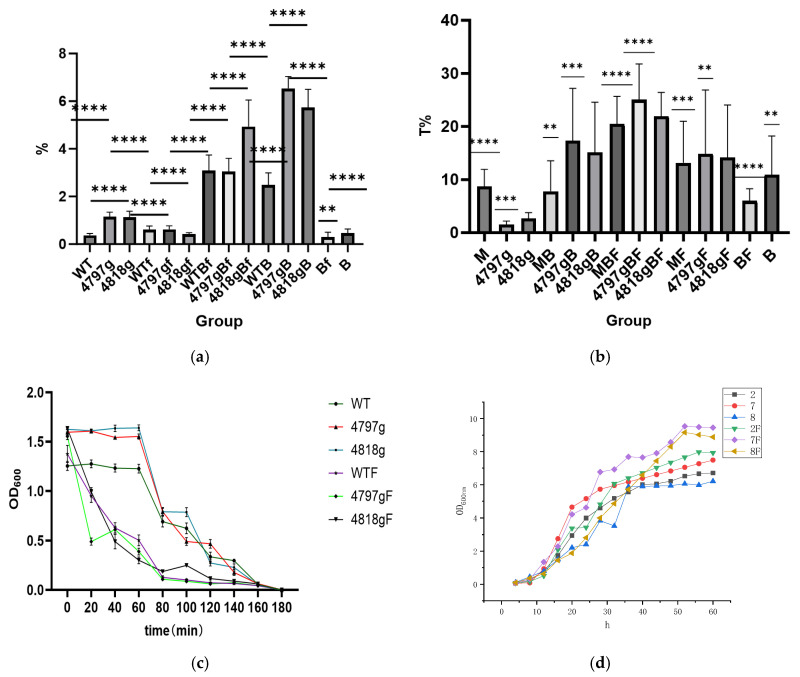
Basic functional analysis of overexpression strains. Note: (**a**) is the result of MTT staining, the *M. bicuspidata* group WT, the 4818 gene overexpression group 4818g, the 4797 gene overexpression group 4797g, the Fe ion group (WTF, 4818gF, 4797gF), the hemocyte group (WTB, 4818gB, 4797gB, B), and the co-stress group (WTBF, 4818gBF, 4797g, BF). (**b**) is the film-forming ability assay, the *M. bicuspidata* group M, the 4818 gene overexpression group 4818g, the 4797 gene overexpression group 4797g, the Fe ion group (MF, 4818gF, 4797gF), the hemocyte group (MB, 4818gB, 4797gB, B), and the co-stress group (MBF, 4818gBF, 4797g, BF). (**c**) is the flocculation ability assay, the *M. bicuspidata* group WT, the 4818 gene overexpression group 4818g, the 4797 gene overexpression group 4797g, the Fe ion group (WTF, 4818gF, 4797gF). (**d**) is the growth curve of the overexpression strains, the *M. bicuspidata* group 2, the 4818 gene overexpression group 8, the 4797 gene overexpression group 7, the Fe ion group (2F, 8F, 7F). “**” means *p* < 0.01, “***” means *p* < 0.001, “****” means *p* < 0.0001.

The MTT toxicity detection kit measured overexpression strains and found that after the addition of blood cells, two overexpression strains’ toxicity increased, with the addition of Fe ions, two strains did not differ, and blood cells and Fe ions added at the same time improved 4818 more (Figure 14a); overexpression strains in the case of co-incubation of blood cells the strongest film-forming ability, with the addition of Fe ions, the film-forming ability is stronger than the control (Figure 14b); the flocculation ability of two overexpression strains’ flocculation rate is greater than wild bacteria, with the addition of Fe ions, all strains’ flocculation rate was improved. The flocculation ability of the overexpression strains was greater than that of the wild bacteria, and the flocculation rate of all strains was enhanced by the addition of Fe ions (Figure 14c). As can be seen from the figure, the growth of the Fe ion group was faster, in which the 4797 overexpression strain with Fe ion was the fastest, and in the group without Fe ion, the 4797 overexpression grew faster, but the 4818 grew slower (Figure 14d).

### 3.9. Overexpression of the Sense of Return

**Figure 15:**
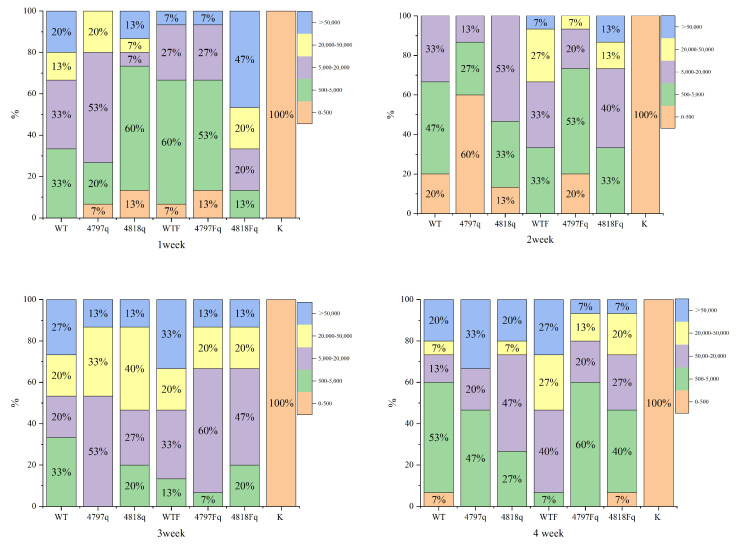
Percentage stacked bar chart for infection experiments with overexpression strains. Note: the *M. bicuspidata* group WT, the 4818 gene overexpression group 4818g, the 4797 gene overexpression group 4797g, the Fe ion group (WTF, 4818gF, 4797gF), the K is blank control; 0–500, 500–5000, 5000–20,000, 20,000–50,000, >50,000 are the range of colony counts.

The results of the back-sensing experiment showed that the overexpression strains were stronger than the wild bacteria in the first three weeks, but the wild bacteria were stronger than the overexpression strains in the fourth week; the overall infection ability of the group with the addition of Fe ions was stronger than that of the group without the addition of Fe ions, and the overexpression strains were more obvious (Figure 15).

## 4. Discussion

Transcriptome sequencing as a technology for gene expression analysis research, with the development of science and technology, is widely used in various fields of research. It can detect the sum of all RNAs transcribed by a specific cell or tissue in different growth and development periods or different functional states, and through the database search and analysis of the depth of mining information [24,25]. In this experiment, the pre-infection experimental findings, the special node of 8 h was selected, when the mitomycetes yeast and blood cells exercised their peak functions, and the experiment was set up with three treatment groups of blood cell stress, Fe ion stress, and co-stress to simulate the condition of blood cells infected by *M. bicuspidata* after infection of river crabs. Fe ions were added to simulate that the local nurseries in Panjin use well saline water, which contains a large number of Fe ions; the concentration of Fe ions in well saltwater added to the nursery process can exceed 10 mg/L. The concentration of Fe ions in overwintering river crab water can also exceed 2 mg/L [1]. The addition of Fe ions was to simulate whether Fe ions in the aquaculture environment would increase the infectivity of *M. bicuspidata*.

Transcriptome data analysis showed that the correlation of these sequencing data was good, the percentage of Q30 bases was not less than 93.74%, and the R-value was more than 0.95, indicating data reliability. Hematocrit significantly activated base excision repair (−log10(P) = 7.58) and ribosome biogenesis-related pathway (KEGG). The extremely high enrichment of base excision repair suggests that hemocytes may attack the fungal genome by generating reactive oxygen species (ROS) or releasing DNA damage factors (e.g., antimicrobial peptides), forcing the yeast to initiate DNA repair mechanisms to maintain genome stability. Meanwhile, enrichment of ribosome-associated genes (“cytosolic ribosome”, “ribosomal subunit”) (GO) suggests that yeast may be used to generate stress proteins (e.g., molecular chaperones) or repair enzymes by enhancing protein synthesis capacity or repair enzymes [26]. In addition, downstream of propanoate (−log10(P) = 1.32) lipid metabolism, propionic acid is converted to succinyl coenzyme A, which then enters the tricarboxylic acid cycle, providing energy to support rapid adaptation in host/pathogen interactions [27]. The MAPK signaling pathway is involved in the regulation of a variety of fundamental cellular processes, such as cellular growth, differentiation, survival, apoptosis, migration, inflammation, and environmental stress response [28]. In this study, the cellular immune MAPK pathway was significantly enriched in *M. bicuspidata* to adapt to hemocytes under hemocyte stress as an environmental processing process that would be significantly enriched in response to cellular stress, in line with the study of Ke-Xin Jianl [29].

The transcriptome of *M. bicuspidata* was significantly altered after the addition of iron ions, and its differentially expressed genes were mainly enriched in functional categories and pathways related to iron homeostasis and response to oxidative stress [30]. Among the biological processes (BPs), the significantly enriched yeast for glucose metabolism regulation may support the activity of ferric ion transmembrane transport (e.g., Ftr1 iron transporter protein) by enhancing the glycolytic pathway for rapid generation of ATP, as well as providing an energy base for the antioxidant defense system [31]. Another significantly enriched biological process, the phenylacetate catabolic process, may be associated with the degradation of secondary metabolites or the mitigation of cellular damage by scavenging iron-induced toxic metabolic intermediates [32]. At the molecular function (MF) level, the enrichment of oxidoreductase activity (e.g., “codonosuctase activity”) may reflect the maintenance of intracellular redox homeostasis in yeast via iron/sulfur cluster synthesis or ROS-scavenging enzymes (e.g., superoxide dismutase) in response to iron overload-induced Fenton’s reaction [33,34].

KEGG pathway analysis further revealed the global effect of iron ion addition on the yeast metabolic network. Glutathione metabolic reactions can be classified as glutathione conjugation, glucosylation, methylation, or detoxification of reactive oxygen species [35], and the enrichment of glutathione metabolism (glutathione metabolism, −log10(P) = 0.88) in this experiment suggests that yeast glutathione (GSH) synthesizes and reduces the cyclic system to neutralize ROS, such as hydroxyl radicals, generated by iron overload. This mechanism is consistent with the conserved strategy of fungi to cope with oxidative stress [36]. Meanwhile, activation of the pentose phosphate pathway (pentose phosphate pathway, −log10(P) = 0.71) can maintain the reduced state of GSH and enhance antioxidant defenses by generating NADPH [37]. In terms of energy metabolism, the enrichment of ubiquinone terpenoid quinone biosynthesis (ubiquinone biosynthesis, −log10(P) = 1.27) suggests that coenzyme Q synthesis in the mitochondrial electron transport chain may be regulated by iron to balance oxidative phosphorylation and ROS generation [38]. Mild activation of the autophagy (autophagy-yeast, −log10(P) = 0.34) and proteasome (proteasome, −log10(P) = 0.4) pathways may be synergistically involved in iron homeostatic regulation: autophagy releases stored iron by degrading ferritin aggregates, while the proteasome is responsible for scavenging iron-induced oxidative damage proteins, preventing the accumulation of misfolded proteins [39,40].

The co-stress group GO resulted in significant changes in ribosome-related components, functions, and processes in yeast cells. The yeast transcriptome showed a unique adaptive strategy under the combined treatment of iron ions and hemocytes. KEGG analysis revealed a significant enrichment of ribosome biogenesis (ribosome biogenesis, −log10(P) = 1.3) and non-homologous end-joining (non-homologous end-joining, −log10(P) = 0.72) pathways, suggesting that under combined stress, yeast must cope with both protein synthesis demands (e.g., stress proteins) and DNA double-strand break repair [40]. The enrichment of the ATPase complex in GO analysis (e.g., “proton-exporting ATPase activity”) suggests that the vesicular membrane V-ATPase may coordinate iron storage with immune escape by regulating intracellular pH and iron ion distribution [41]. In addition, activation of pyruvate metabolism (pyruvate metabolism, −log10(P) = 0.87) may enhance energy supply through the tricarboxylic acid cycle to support survival competition in the host environment [27].

Iron ions are essential trace elements for fungal growth and metabolism and are involved in the synthesis and functional regulation of ribosomes. The significant enrichment of ribosome-related components and functions in this study demonstrated the important role of iron ions in regulating ribosome dynamic homeostasis and protein synthesis in yeast cells [42]. The results of KEGG enrichment analysis further supported the results of GO analysis, and iron ion treatment had a significant effect on the process of gene information processing in yeast cells, especially the significant enrichment of ribosomes (ribosome)-related pathways, suggesting that iron ions regulate gene expression in yeast cells, protein synthesis, and ribosome biosynthesis in yeast cells. As a cofactor, iron is involved in a variety of enzymatic reactions, and maintaining its homeostasis is essential to ensure normal metabolism and physiological functions of fungal cells [43]. This interaction was further supported by the enrichment of the MAPK pathway (MAPK pathway, −log10(P) = 0.88) in the co-stress group, which often mediates the transduction of external stress signals (e.g., oxidative stress, osmotic pressure) in fungi. Lin Wei obtained significant regulation of genes at all levels of the MAPK pathway when studying the effects of cadmium ion tolerance in yeast, in which the hypertonic glycerol pathway (HOG) and the cell wall integrity pathway (CWI), the two core components of the MAPK cascade system, may be related to the regulation of cadmium ion tolerance, which is similar to this work [44].

During data analysis, two CFEM genes were found to be up-regulated in the hemocyte stress and co-stress groups and down-regulated in the Fe ion stress group. CFEM genes play a key role in Fe ion uptake, especially in the host environment where access to Fe ions is critical for the survival and the pathogenicity of the fungus [45]. Vivek Kumar SRIVASTAVA, in his study of *C. albicans* Fe homeostasis, demonstrated the involvement of Fe ion uptake in adhesion, survival, and virulence in the host and verified the involvement of CFEM genes in the process of Fe uptake [46]. To determine whether the CFEM genes play a role in the mitogenic process, we constructed CFEM gene overexpression strains, and the overexpression results showed that the CFEM gene overexpression strains were significantly enhanced in flocculation, membrane formation, and Fe ion uptake and infection, which proved that the two CFEM genes we found were involved in the processes of flocculation, membrane formation, and Fe ion uptake and infection and that it was a multifunctional gene. Zhang isolated a CFEM protein in *Staphylococcus griseus* and significantly up-regulated it during the early stage of bean leaf infection, and used Agrobacterium osmosis to induce chlorosis in Benjamin’s tobacco leaves, but overexpression of BcCFEM1 did not result in any observable phenotypic changes [47]. Dan Wang also found four CFEM proteins in *Candida albicans*, Csa1, Csa2, Rbt5 and Pga7, Rbt5, and Pga7, were shown to play a key role in biofilm formation and virulence by acquiring heme from the binding site of aspartic acid (Asp, D) residues within the structural domain of CFEM [48], which is consistent with our results in hemocyte adhesion, MTT staining experiments with overexpressing strains in back-sensing experiments.

In summary, *M. bicuspidata* significantly enhanced biofilm formation (as determined by saffron staining), flocculation rate (increased by 20%), and infection toxicity (as defined by MTT toxicity, increased by 35%, and back-sensing infection rate, increased by 40%) through overexpression of the CFEM genes (4797/4818). This activation provided a precise control strategy. Targets such as iron uptake and biofilm formation can be blocked by developing CFEM protein inhibitors, and the iron concentration in the culture water can be strictly controlled. However, there are limitations, such as insufficient in vivo validation and a lack of environmental factors, such as temperature and salinity. There is also a lack of long-term re-sensitization experiments and gaps in inhibitor safety. Future studies should focus on the dynamic pathogenic mechanism, analyze how environmental factors regulate CFEM, and promote the safety evaluation of peptide vaccines based on the conserved CFEM domain (60 aa) and iron-chelating agents. This will enable us to target and control CFEM. Synergistic prevention and control through intervention and water management will ensure the sustainable development of river crab aquaculture.

## 5. Conclusions

The analysis of *Metschnikowia bicuspidata* genome sequencing revealed the fungus’s diverse adaptive mechanisms. Under stress conditions, the fungus activates DNA repair and protein synthesis, resisting immune attacks, and iron stress activates the pentose phosphate pathway and glutathione metabolism. Yeast responds to multiple stressors through the stress response pathway, non-homologous end-joining, and vesicular V-ATPase-regulated iron distribution. Experiments revealed that overexpressing CFEM genes enhances virulence. The addition of iron ions enhances adhesion, biofilm, and flocculation, confirming that CFEM drives infection by regulating iron uptake and biofilm formation. This finding is important for understanding the function of the *Metschnikowia bicuspidata* CFEM gene and the pathogenic mechanism of Fe ions in “milk diseases”. It also lays the theoretical groundwork for developing a strategy to prevent and treat “milk diseases”.

## Figures and Tables

**Table 1:** The primers for CFEM cloning used in this study.

Primer Name	Sequence (5′ to 3′)
4797	F tacttgaacacactaggatccATGTTGTATTACCTCCCTGTCTTGAC
	R tggcctatgtctagaggatccTCAGACTATAAGAGATAATATGATGACTGAAA
4818	F tacttgaacacactaggatccATGTTGTCTGCTTTTATTCCTCTCG
	R ggcctatgtctagaggatccTTAGAGGATCAAAGCGACACCTG

**Table 2:** The primers for RT-qPCR used in this study.

Primer Name	Sequence (5′ to 3′)
4797f	TGTGGCTTCATGTACGGCTG
4797r	GCAGGCATCATCCACAAGTTGG
4818f	GCCCGTTTGTGCGCAAGCAT
4818r	GGAGGCAAAGTTGCTCATCACGCA
β-actin-f	CAGGAAATGACCACTGCCGC
β-actin-r	CGGAACCTCTCATTGCCGA
2216f	TCTTTGGCGGCATCGACACA
2216r	CCAAAGAAGTGCCAGAGTCCAA
2333f	ATACGAGGGCTCCTTGACGA
2333r	GACAAGTGCCAACGAGGTTC
2655f	ACTCGCTGTTCTTCACCAACC
2655r	R GATGGCAGACGGGAGGTAG

**Table 3:** Quality control of transcriptome data.

Sample	Raw Read	Raw Bases	Q20%	Q30%	Trimmed Read	Trimmed Bases	Useful Read%	Useful Bases%	tRNA Pseudogene	tRNA	Protein Coding
M1	49,272,578	7,440,159,278	97.67	93.75	48,380,248	7,287,767,821	98.19	97.95	11	870	18,566,434
M2	44,356,168	6,697,781,368	97.67	93.74	43,549,464	6,563,612,926	98.18	98	6	989	16,701,740
M3	54,429,536	8,218,859,936	97.1	94.78	53,451,518	8,027,390,053	98.2	97.67	14	975	20,542,974
MB1	37,283,104	5,629,748,704	97.73	93.87	36,616,604	5,518,129,766	98.21	98.02	2	714	14,086,880
MB2	37,386,686	5,645,389,586	97.53	93.48	36,628,310	5,519,487,920	97.97	97.77	1	748	14,056,547
MB3	48,731,056	7,358,389,456	97.69	93.78	47,861,688	7,215,326,824	98.22	98.06	3	1036	18,294,879
MF1	42,020,984	6,345,168,584	97.68	93.79	41,253,050	6,212,916,494	98.17	97.92	21	567	15,898,276
MF2	46,345,586	6,998,183,486	97.72	93.86	45,531,436	6,860,178,616	98.24	98.03	20	594	17,527,590
MF3	45,440,904	6,861,576,504	97.86	94.18	44,687,612	6,729,584,867	98.34	98.08	23	691	17,120,775
MFB1	39,501,182	5,964,678,482	97.81	94.08	38,847,978	5,853,839,115	98.35	98.14	11	403	15,003,958
MFB2	39,273,624	5,930,317,224	97.68	93.78	38,577,900	5,809,783,618	98.23	97.97	9	455	14,933,104
MFB3	42,929,950	6,482,422,450	97.78	94.04	42,184,496	6,355,544,543	98.26	98.04	7	478	16,296,246

Note: Sample: name of sample; Reads No.: total number of reads; Bases (bp): total number of bases; Q30 (bp): total number of bases with base recognition accuracy above 99.9%; Q20 (%): percentage of bases with base recognition accuracy above 99%; Q30 (%): percentage of bases with base recognition accuracy above 99.9%. Percentage. *M. bicuspidata* control (sterile medium, M/CK), hemocyte stress control (MB/T1), Fe^3+^ ion control (1 mM FeCl_3_, MF/T2), and co-stress group (MBF/T3).

## Data Availability

All the raw sequencing reads from this study have been submitted to the NCBI Sequence Read Archive (SRA) under BioProject accession numbers PRJNA910793.
